# Optimal Heart Rate Modulation Therapy Using Ivabradine in Individuals With Acute Heart Failure

**DOI:** 10.1002/clc.70248

**Published:** 2025-12-25

**Authors:** Naoya Kataoka, Teruhiko Imamura

**Affiliations:** ^1^ Second Department of Internal Medicine University of Toyama Toyama Japan

**Keywords:** echocardiography, hemodynamics, HFrEF

To editor:

1

Ivabradine demonstrates notable efficacy in ameliorating clinical outcomes among patients with chronic systolic heart failure, yet its therapeutic implications in the context of acute heart failure remain a subject of contention. In a comparative analysis conducted by Tsai et al. the investigators evaluated clinical ramifications following the initiation of ivabradine in individuals experiencing acute decompensated heart failure, juxtaposed with a cohort of contemporaneously matched counterparts devoid of ivabradine exposure [1]. The study discerned a consistent and positive therapeutic impact of ivabradine, with no discernible divergences of statistical significance observed between the groups treated with ivabradine and those without, particularly concerning incidences of heart failure‐related hospitalization and cardiovascular mortality.

Ivabradine finds clinical indication in individuals exhibiting intolerance to beta‐blockers, an established cornerstone therapy pivotal for effecting reverse remodeling and fostering enhanced clinical outcomes in patients with systolic heart failure [2]. The authors' investigation revealed a substantial proportion of participants characterized by relatively preserved blood pressure and an absence of significant bradycardia, with approximately 30% of them concurrently administered calcium blockers [1]. It is imperative for the authors to elucidate the reasons underpinning beta‐blocker intolerance within their study cohort. Of note, acute heart failure does not categorically mandate the complete cessation of beta‐blocker therapy [3].

The therapeutic potential of ivabradine extends beyond mere symptom alleviation, encompassing the facilitation of optimal hemodynamics conducive to the judicious titration of beta‐blockers [4]. The study lacks clarification regarding whether the authors undertook a robust up‐titration of heart failure medications subsequent to the initiation of ivabradine [1]. This aggressive approach could potentially yield indirect improvements in clinical outcomes by virtue of ivabradine's stabilizing influence on hemodynamics.

The determination of an optimal heart rate in individuals grappling with acute heart failure remains an unresolved quandary. A suboptimal reduction in heart rate may inadvertently diminish cardiac output and exacerbate hemodynamic instability. To address this concern, our team advocates a novel methodology for heart rate optimization. Specifically, we propose the minimization of overlap between the E‐wave and A‐wave in Doppler echocardiographic trans‐mitral flow during ivabradine therapy as a strategy to maximize cardiac output [4]. The authors should explicate their rationale for selecting the target heart rate in their study, shedding light on the considerations that informed this crucial parameter.

## Disclosure

The authors have nothing to report.

## Conflicts of Interest

The authors declare no conflicts of interest.

## Data Availability

Data sharing is not applicable to this article as no data sets were generated or analysed during the current study.
